# Caregiving Quality Across Development and Secure Base Knowledge among Adolescents with a History of Institutional Care

**DOI:** 10.1007/s42844-025-00183-2

**Published:** 2025-10-26

**Authors:** Megan M. Hare, Elia Psouni, Katherine L. Guyon-Harris, Kathryn L. Humphreys, Nathan A. Fox, Charles A. Nelson, Charles H. Zeanah

**Affiliations:** 1 Department of Psychiatry and Behavioral Sciences, Tulane University School of Medicine, New Orleans, LA 70112 USA; 2 Department of Psychology, Lund University, Lund, Sweden; 3 Department of Pediatrics, University of Pittsburgh School of Medicine, Pittsburgh, PA 15224 USA; 4 Department of Psychology and Human Development, Vanderbilt University, Nashville, TN 37203 USA; 5 Department of Human Development and Quantitative Methodology, University of Maryland, College Park, MD 20742 USA; 6 Boston Children’s Hospital, Harvard Medical School, Cambridge, MA 02115 USA; 7 Harvard Graduate School of Education, Cambridge, MA 02138 USA

**Keywords:** Attachment, Deprivation, Secure base script, Caregiving quality, Institutionalization, Adolescence, Foster care

## Abstract

Secure base scripts are implicit schemas that guide perceptions, responses, and interactions by encapsulating expectations of support from attachment figures during distress. The current study investigated the impact of early institutional rearing and the causal effects of high-quality foster care following early deprivation on secure base scripts in adolescents who had experienced severe early deprivation. We evaluated whether caregiver quality, assessed 5 times from ages 30 months to 12 years, predicted secure base scripts at age 16 years, assessed with the Secure Base Script Test (SBST). We analyzed data from 121 participants of the Bucharest Early Intervention Project, comprising institutionalized children who were randomized to care as usual (CAUG) or to high-quality foster care (FCG). We also included a group of never-institutionalized comparison children (NIG). The NIG exhibited higher SBST scores compared to children with a history of institutional rearing, highlighting the detrimental effects of early deprivation. Contrary to expectations, no statistically significant group differences emerged between FCG and CAUG on SBST scores. However, individual differences in caregiving quality, regardless of group, predicted SBST scores, with caregiving at ages 8 and 12 years emerging as stronger predictors than earlier assessments. Thus, that while early deprivation can have lasting effects on secure base script development, caregiving quality in middle childhood may play a particularly important role in shaping attachment-related representations in adolescence.

**Supplementary Information:**

The online version contains supplementary material available at 10.1007/s42844-025-00183-2.

Attachment theory suggests that early caregiver–child relationships serve as the foundation for a child’s socioemotional development, shaping expectations about support, safety, and interpersonal trust throughout the lifespan (Bowlby, 1969; 1979). A central construct within this framework is the secure base script, a cognitive schema that organizes expectations about how attachment figures respond during times of distress (Waters & Waters, 2006). These scripts, which are thought to emerge from early experiences, purportedly guide how individuals perceive, interpret, and navigate relationships across the lifespan. Although secure base scripts have been linked to attachment security and psychosocial functioning (Dykas et al., 2006; Psouni & Apetroaia, 2014), relatively few studies have examined these scripts in adolescents with histories of early adversity (e.g., Nivison et al., 2021), and to our knowledge, none have done so in the context of institutional rearing.

Emerging research, however, supports links between early caregiving experiences and later secure base script knowledge. For instance, using a subsample of the NICHD Study of Early Child Care and Youth Development, Steele et al. (2014) found that the proportion of toddler and preschool age assessments in which a child’s relationship was classified as secure was positively associated with secure base script knowledge at age 18 years. This work also demonstrated that maternal and paternal sensitivity across development contributed to secure base script formation in late adolescence, suggesting that attachment-related representations may be shaped both by early and later caregiving experiences. Therefore, understanding how such representations form is especially important in populations exposed to early psychosocial deprivation, where caregiving has been disrupted or compromised during sensitive periods of development.

Extensive research has documented the negative impact of institutional rearing, marked by social and emotional deprivation, on developmental outcomes, including organization and security of attachment, emotion regulation difficulties, and cognitive delays (Smyke et al., 2002; Zeanah et al., 2005; Nelson et al., 2007). Within this context, caregiver sensitivity and stability have emerged as robust predictors of more adaptive outcomes, including the development of secure and organized attachments and improved social functioning (Bakermans-Kranenburg et al., 2011; Guyon-Harris et al., 2021). For example, within the same sample as the current study, Smyke et al. (2010) showed that caregiving quality predicted more organized attachments at 42 months. However, questions remain about the persistence and plasticity of these effects over time. Specifically, does early caregiving exert a lasting impact on adolescent attachment representations, or are more recent caregiving experiences more influential?

Some perspectives emphasize the enduring impact of early attachment relationships (e.g., Roisman et al., 2005), consistent with Bowlby’s (1973) idea of internal working models that guide future relationships in a relatively stable manner. Others argue that these models are open to revision and sensitive to more recent caregiving contexts, particularly during middle childhood and adolescence, when children may experience new caregiving relationships or significant developmental shifts (Lewis et al., 2000; Weinfield et al., 2000). Yet empirical efforts to disentangle the timing of caregiving influences are limited, as most children remain with the same caregivers across development, making it difficult to determine whether early or later caregiving exerts greater influence on attachment-related outcomes.

The Bucharest Early Intervention Project (BEIP), a randomized controlled trial of foster care as an alternative to institutional care for abandoned children, offers an opportunity to explore whether and when in development caregiving quality is associated with secure base scripts among adolescents who experienced severe early deprivation. In the present study, we had three aims. First, we examined the association between early psychosocial deprivation and secure base scripts in adolescence (i.e., age 16 years). We expected that children who experienced deprivation associated with institutional rearing would demonstrate poorer secure base knowledge, as reflected in their secure base scripts assessed by word-prompted narratives, compared to never institutionalized community children. Second, we tested the effects of high-quality foster care following early deprivation on secure base scripts. We predicted that children randomized to foster care would demonstrate scripts reflecting more secure base knowledge compared to children who were randomized to care as usual, who as a group experienced more prolonged institutional exposure. Third, we explored whether caregiver quality, assessed 5 times from ages 30 months to 12 years, was associated with secure base knowledge at age 16 years, and whether caregiving quality at some ages had a stronger predictive value than others. This exploratory investigation aimed to shed light on the relative importance of early versus more recent caregiving quality in influencing secure base scripts during adolescence.

## Method

### Setting

The BEIP includes a sample of 136 children (aged 6 to 30 months, mean age = 22 months) recruited from all 6 institutions for young children in Bucharest. Half of the children (*n* = 68) in institutional care were randomized to a high-quality foster care intervention, resulting in two groups: the foster care group (FCG) and a group exposed to care as usual (CAUG). Children in the FCG were placed into foster care families following randomization; however, not all children remained in their initial foster placements, and some experienced changes in caregiving arrangements due to disruptions, transitions, or reunifications over the course of the study. Similarly, children in the CAUG group often experienced multiple caregiver changes within institutional settings and, in some cases, transitions to family care later in development.

**Fig. 1:**
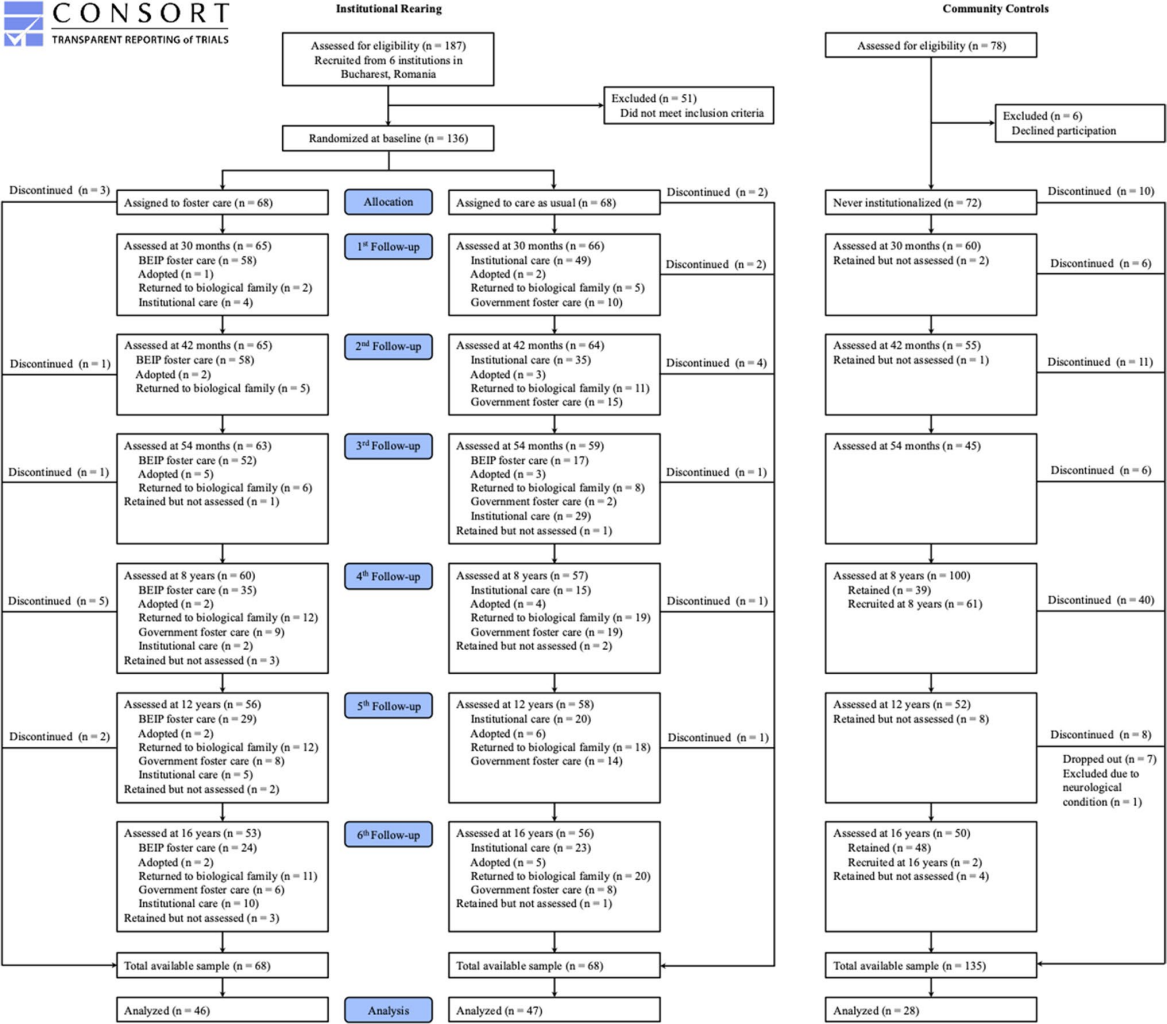
Consort diagram

Additionally, at the initial assessment, 72 children with no history of institutionalization (NIG) who were age and sex matched to the other groups were recruited from public pediatric clinics (see Fig. 1). Although additional NIG participants were recruited at age 8 years to supplement the original community comparison sample, these later-enrolled children were not included in the present analyses to ensure consistency in group comparisons and reduce the potential for sample heterogeneity. After approval by the institutional review boards of the three principal investigators (MASKED), and by the local Commissions on Child Protection in Bucharest, the study started in collaboration with the Institute of Maternal and Child Health of the Romanian Ministry of Health. We obtained signed consent from each child's legal guardian as per Romanian law at every time point (i.e., 30 months, 42 months, 54 months, 8 years, 12 years, and 16 years). We obtained written assent from each child for each procedure at ages 8, 12, and 16 years (unless the child had intellectual disabilities, in which case they gave verbal assent). The children who had never been placed in an institution also gave written assent and their legal guardians (parents) completed signed consents. Further details about the original sample are available elsewhere (Zeanah et al., 2003).

### Participants

The current study includes 121 participants who had complete SBST data at age 16 years (FCG = 46, CAUG = 47, NIG = 28). To maintain consistency in sampling, we excluded NIG participants who were newly recruited at the age 8 (*n* = 19) follow-up and included only those who were part of the original baseline cohort. The participants were 50% female and 50% male and underwent assessments at a mean age of 16.52 years (*SD* = 0.72) as part of longitudinal follow-up of BEIP. Participants were mostly Romanian (61%), while 27% identified as Rroma, and 12% did not provide information on ethnicity. There were no differences in terms of age, gender or ethnicity between participants included in the analysis and those who discontinued participation or did not complete the assessment at age 16 (*p* >.05).

### Measurements

#### Secure Base Scripts

Secure base knowledge was measured at age 16 years using the SBST (Psouni & Apetroaia, 2014). The SBST requires participants to create four stories, each guided by a story title and a set of word-prompt outlines meant to bring to mind age-appropriate attachment-related situations (e.g., “Math test” story-title and word-prompt outline which implies a story where a child must talk with their caregiver about a math test that did not go well). Each outline consists of 12 words presenting the story premise and main characters, a crisis involving the main character, and implying interactions with caregivers/friend and resolution. Two sets imply storylines involving secure base interactions with parents (Math test, Accident), while two sets feature interactions between best friends (Troubles at school, Moves away). A fifth, neutral theme is included as an initial “warm-up,” to familiarize participants with the test procedure and provide an opportunity to practice telling a story with inspiration from word-prompts.

The SBST was deemed preferable to other, similar measures of scripted secure base knowledge, for two main reasons. First, its outlines transpire as relatively culturally neutral, compared to the outlines included in a word-prompt narrative attachment instrument for adolescents (Dykas et al., 2006), which are rather culturally specific (e.g., acne troubles, a tennis match) and may lack ecological validity in the target population. Second, while these outlines in the measure for adolescents (Dykas et al., 2006) are also specifically mother-related (acne/haircut, party) and father-related (tennis match, studying for an exam), the SBST does not rely on assumptions of two-parent caring environments or a priori assumptions of certain types of gendered behaviors in parenting. Instead, the SBST uses the compound “mother/father” to prompt for caregiver, leaving it up to the respondent to choose the exact word and caregiving figure to incorporate in their narrative. Notably, while the two word-prompt narrative tests (SBST and adolescent) differ in terms of story titles/content of the story stems, there are no differences in how the narratives are scored.

Participants were asked to tell the best story they could using the prompt words as a guide. If necessary, during the warm-up story, the interviewer provided respondents with further guidance about the procedure and instructions to ensure their understanding (e.g., asking an open-ended question or starting a sentence to draw the story out further, encouraging the child to continue). The four test outlines were then presented, counterbalanced for order. Transcribed narratives from the attachment-related outlines were scored on a seven-point scriptedness scale according to a scoring manual (Psouni & Apetroaia, 2014), reflecting how much they resemble a prototypical secure base script, a story that reflects a crisis resolution based on elaborate, supportive interactions rich in emotional exchange. High scores denote narratives that contain rich knowledge of secure base interactions and are complete and coherent. Co-protagonists (caregivers or close friends) are described as attentive and promptly providing practical and emotional support in difficult situations, contributing to resolution of the difficulty and affect regulation. While scores of “4” denote narratives that involve just sufficient secure base content to suggest a rudimentary script (Psouni & Apetroaia, 2014), stories with lower scores contain few or no elements of secure base interaction, and may contain sequences of events without emotional content. Supplementary Table S1 presents examples of high scriptedness and low scriptedness narratives.

Narratives were coded organized by story title (Math test, Accident, Troubles at school, and Moves away) in a manner that blinded which stories originated from the same child, or the children’s care history (care as usual vs. foster care). One senior reliable SBST coder with extensive experience in coding attachment script narratives (Attachment Script Assessment/ASA: Waters & Waters, 2006, and SBST) and other attachment measurements (2nd author) coded all the material. A second reliable SBST coder, formally trained and tested reliable one year prior to the study (research assistant), independently coded 35% of the material (50 cases). Each of the four title scores (Math test, Accident, Troubles at school, and Moves away, respectively) demonstrated strong inter-rater reliability (ICC range =.94–.96). A mean total score was derived, wherein higher scores indicate greater SBS knowledge (Cronbach’s α =.85).

### Caregiving Quality

Caregiving quality was assessed at 30 and 42 months using the Observational Record of the Caregiving Environment (NICHD Early Child Care Research Network, 1996). Children were videotaped interacting with their preferred caregiver for 1½ h in their natural care environment. Coders rated five qualitative scales, detachment, flat affect, positive regard for the child, sensitivity, and stimulation of development, on Likert scales from 1 (not at all characteristic) to 4 (highly characteristic). The caregiving-quality score was then computed as the mean of these five scales, producing a single composite score ranging from 1 (low quality) to 4 (high quality). Coders underwent extensive training using a standardized manual, including orientation to all items, practice with 10 reliability tapes, and discussion-based resolution of discrepancies. These training tapes were drawn from both pilot data and a local U.S. community sample to ensure range and relevance. Inter-rater reliability was maintained throughout data collection via double-coding of 40% of the tapes, yielding high reliability (ICC range =.88–.99, *M* =.95). Scale reliability was excellent (Cronbach’s α =.86). Importantly, all coders were blind to group assignment (CAUG, FCG, NIG) during coding procedures.

At 54 months, 8 years, and 12 years, caregiving quality was assessed using global ratings made by two BEIP staff members based in Romania, each of whom was familiar with the children and their caregiving contexts at each age. These raters did not conduct joint visits; rather, they based their ratings on a shared pool of information gathered across numerous hours of observations over time, including both laboratory visits and informal interactions with the family (Colich et al., 2021). Thus, although they may have had separate experiences with the caregiver–child dyads, their evaluations drew from overlapping and cumulative observational exposure. Ratings considered multiple aspects of caregiving, such as the fulfillment of basic needs, consistency and quality of emotional care, caregiver availability, and the overall caregiver–child relationship. Ratings were made on a 5-point ordinal scale: (a) Dangerous, (b) Unacceptable, (c) Marginal, (d) Mixed, and (e) Acceptable. Inter-rater reliability was excellent (ICC =.93), and the scores from the two raters were averaged to generate a caregiving quality score ranging from 1 to 5. Table 1 presents the means and standard deviations of caregiving quality scores at each assessment age, separately for each group.

**Table 1:** Ratings of caregiving quality across all time points

	30 months	42 months	54 months	96 months	144 months
Care as Usual Group	M = − 0.40 R = − 2.98, 1.62 SD = 1.05 *n* = 36	M = − 0.22 R = − 2.28, 1.34 SD = 0.97 *n* = 35	M = − 0.70 R = − 2.24, 0.71 SD = 1.18 *n* = 46	M = − 0.75 R = − 2.74, 0.79 SD = 1.06 *n* = 45	M = − 0.67 R = − 2.47, 0.78 SD = 1.03 *n* = 47
Foster Care Group	M = 0.13 R = − 2.63, 1.63 SD = 0.90 *n* = 41	M = 0.17 R = − 1.93, 1.86 SD = 1.00 *n* = 40	M = 0.42 R = − 1.14, 0.71 SD = 0.48 *n* = 41	M = 0.22 R = − 2.30, 0.79 SD = 0.78 *n* = 44	M = 0.15 R = − 2.06, 0.79 SD = 0.91 *n* = 46
Never Institutionalized Group	M = 0.40 R = − 1.57, 1.98 SD = 0.89 *n* = 23	M = 0.40 R = − 1.54, 1.51 SD = 1.00 *n* = 24	M = 0.54 R = −0.77, 0.81 SD = 0.36 *n* = 22	M = 0.56 R = −0.98, 0.79 SD = 0.54 *n* = 26	M = 0.64 R = −0.94, 0.79 SD = 0.36 *n* = 23

*M* mean, SD standard deviation, R range, *n* number of available data

As there were two different caregiving measures, all variables were z-scored for interpretability. Statistically significant differences (i.e., *p* <.05) were found across all time points such that the foster care group had higher caregiving quality compared to the care as usual group. Statistically significant differences (i.e., *p* <.05) were found across all time points, except 42 months, such that the never institutionalized group had higher caregiving quality compared to the ever institutionalized group

### Data Analysis

Analyses were conducted using SPSS 27, while visualizations were created in R. Data included participants with complete SBST data at age 16 years (N = 121; CAUG = 47, FCG = 46, NIG = 28). First, we conducted an independent samples t-test comparing the NIG to all children ever institutionalized (EIG = CAUG + FCG) on their SBST total scores. To address our second aim, we used an intent-to-treat (ITT) analysis to compare SBST scores between children randomized to care as usual (CAUG) and those randomized to foster care (FCG), also using an independent samples *t*-test. To examine the third aim, which focused on the association between caregiving quality and secure base script knowledge, we employed multilevel modeling (MLM**)**. MLM was selected to account for the nested structure of the data (i.e., multiple story ratings nested within each participant). To supplement the primary multilevel models, we also computed Spearman correlations between caregiving quality at each assessment time point and SBST scores.

Assumptions were met to indicate that data was missing at random (*x*^2^ (11) = 12.35, *p* =.340). We also conducted a series of chi-square tests to determine whether missing data on the SBST or caregiving quality assessments at each time point were disproportionately distributed across the three study groups. Results revealed no significant group differences in missingness for SBST scores at age 16, χ2(2) = 1.24, *p* =.540. Similarly, for caregiving quality, missingness at 30 months, 42 months, 54 months, 8 years, and 12 years was not significantly associated with group status (all *p*s >.05). These findings support the assumption that data were missing at random and not systematically related to group assignment. Therefore, missing data for the MLM analyses were handled using the Restricted Maximum Likelihood (REML) method, chosen for its appropriateness in estimating variance components in the context of mixed-effects models. As caregiving quality was assessed via two different measures, all caregiving quality scores were z-scored prior to analyses. Time of caregiving assessment was entered as the child’s age at each assessment in months. The first MLM examined main effects of caregiving quality, while covarying for timing of caregiving assessments on SBST scores. In the second MLM, an interaction of caregiving quality by timing of caregiving assessments was included to examine interactions.

## Results

### Ever- versus Never-Institutionalized Children

As predicted, children who had never been institutionalized (*M* = 4.15, *SD* = 1.02) were rated as having significantly higher SBST scores (*t* = − 2.56,* p* =.013, Cohen’s *d* = − 0.49, 95% CI [− 0.87, − 0.10]) compared to children who had histories of institutional rearing (*M* = 3.69, *SD* = 0.09).

### Intent-to-Treat Effects

In contrast to what we predicted, individuals in the FCG (*M* = 3.79, *SD* = 0.86) did not significantly differ from the CAUG (*M* = 3.60, *SD* = 0.91) in SBST score (*t* = − 1.02, *p* =.311, *d* = − 0.21, 95% CI [− 0.618, 0.197]), though the small difference between groups was in the expected direction.

### Caregiving Quality Across Development on SBST Scores

As shown in Supplementary Table S2, Spearman correlations revealed modest positive associations between caregiving quality and SBST scores at 54 months, 8 years, and 12 years (*r*s =.20–.23, *p*s <.05), whereas caregiving quality at 30 and 42 months was not significantly associated with SBST scores. The first MLM examined caregiving quality effects on SBST scores while accounting for the age of the child at the time of assessment. As predicted, results revealed a significant main effect of caregiving quality (*F* (1, 119) = 5.691, *p* =.017, *b* = 0.023, 95% CI [0.004, 0.042], *SE* = 0.010), such that higher-quality caregiving quality was associated with higher SBST scores. In the first model, the main effect of timing of caregiving assessments on SBST scores was not significant (*F* (1, 119) = 0.048,* p* =.837, *b* =.001, 95% CI [− 0.001, 0.002], *SE* = 0.003), indicating that the temporal aspect did not exert a significant influence on the SBST scores.

**Fig. 2:**
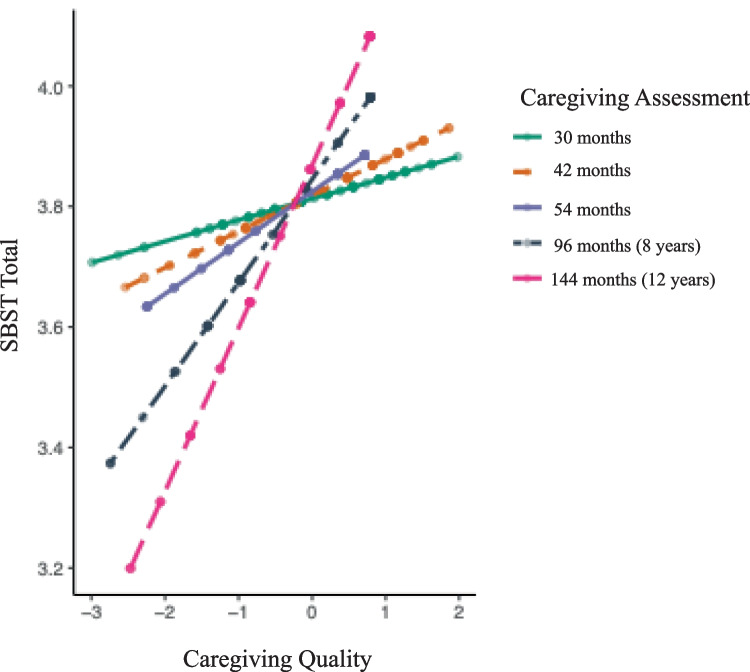
Caregiving quality across time on secure base scripts test total scores at age 16 years

The second model, which included an interaction between caregiving quality and timing of assessments, revealed a significant interaction(*F* (1, 119) = 4.020, *p* =.046, *b* = 0.002, 95% CI [0.001, 0.003], *SE* = 0.009). These results indicate that the influence of caregiving quality on SBST scores varies for caregiving quality at different time points, such that caregiving assessments conducted at ages 8 and 12 years showed a stronger association with SBST scores compared to caregiving quality assessments during early childhood (ages 30, 42, and 54 months; see Fig. 2).

To probe this interaction, we examined the slope of caregiving quality in relation to SBST at each wave. At the 30 month assessment (*b* = 0.22, 95% CI [− 0.158, 0.202], *SE* = 0.092, *p* =.813), 42 month assessment (*b* = 0.041, 95% CI [− 0.150, 0.147], *SE* = 0.091, *p* =.653), and 54 month assessment (*b* = 0.158, 95% CI [− 0.012, 0.133], *SE* = 0.086, *p* =.068), on average, the associations between caregiving quality and SBST scores were not statistically significant. However, at the 8-year assessment (*b* = 0.224, 95% CI [0.067, 0.381], *SE* = 0.080, *p* =.005) and 12-year assessment (*b* = 0.247, 95% CI [0.087, 0.407], *SE* = 0.081, *p* =.003), on average, we observed a positive and statistically significant association between caregiving quality and SBST scores.

## Discussion

Based on data from 121 individuals drawn from a longitudinal study of children with and without a history of institutional care, we examined secure base knowledge in adolescence as a function of care history. We report three main findings. First, individuals with a history of institutional care exhibited significantly poorer secure base knowledge compared to their never institutionalized counterparts from the community. Second, individuals randomly assigned to the FCG did not demonstrate significantly higher secure base knowledge than those assigned to the CAUG. Third, caregiving quality was associated with the adolescents’ secure base scripts. The strength of this association varied over time, and the strongest associations were for assessments of caregiving quality from more recent time points. These findings suggest that early exposure to psychosocially depriving institutional care may influence secure base knowledge in adolescence, while also emphasizing that more proximal caregiving quality may mitigate some of the impact on secure base knowledge in adolescence among those with a history of early deprivation.

Adolescents with a history of early institutionalization were, on average, rated as having significantly lower scripted secure base knowledge at age 16 years, compared to peers who had never been institutionalized. This finding aligns with literature suggesting that early adverse experiences, such as abuse/neglect, may have enduring effects on secure base scripts (Nivison et al., 2021). Individuals with a history of early institutional rearing often face challenges in forming and maintaining secure attachments (Doyle & Cicchetti, 2017; Zeanah et al., 2005). Large institutions like those we studied are characterized by limited caregiver interaction and a lack of consistent, responsive care, often preclude the formation of a secure attachment and corresponding scripted knowledge of secure base for children. Consequently, the enduring effects observed in adolescence, as reflected in lower secure base scripts scores, highlight the persistent effects of early severe deprivation.

In contrast to our expectations, among children exposed to institutional care, those randomized to the foster care intervention did not demonstrate significantly higher secure base scripts scores at age 16 compared to those assigned to care as usual. Previous BEIP findings emphasized the positive effects of stable and supportive caregiving environments on attachment-related outcomes (McGoron et al., 2012; McLaughlin et al., 2012). In fact, children placed in foster care exhibited more secure Strange Situation classifications and higher security ratings than those in the CAUG at 42 months (Smyke et al., 2010). Yet, the current findings suggest that this specific intervention of foster care may not have yielded a significant advantage in terms of secure base scripts, as captured by narrative-based word prompt methods of assessment during adolescence. Although research on the transition from early attachment patterns to later secure base scripts knowledge is limited, one study reported a positive correlation between the proportion of times the child was rated as secure from 15 to 36 months and secure base scripts knowledge at age 18 (Steele et al., 2014). The current findings underscore the complexity of attachment development and the need for ongoing support and interventions that extend beyond early childhood to maintain and build upon initial improvements.

The overall main effect of caregiving quality on secure base scripts scores in adolescence is in line with the theoretical underpinnings of secure base scripts (Waters & Cummins, 2000) and related empirical findings (Krantz et al., 2024; Psouni & Apetroaia, 2014; Steele et al., 2014; Vaughn et al., 2016). However, the association between caregiving quality and secure base scripts scores differed as a function of when caregiving quality was assessed, with caregiving quality in early life less influential than more recent assessments of caregiving quality (ages 8 and 12 years). It may be that the impact of caregiving quality at earlier time points were modified by the intervening effects of subsequent caregiving experiences or environmental changes. Secure base scripts are thought to develop from rudimentary sensorimotor-affective knowledge based on early interactions with caregivers (Nelson, 1986), and it is likely that story completion tasks in early childhood [e.g. Attachment Story Completion Task/ASCT (Bretherton et al., 1990); Manchester Child Attachment Story Task/MCAST (Green et al., 2000)] do capture this rudimentary implicit knowledge. However, episodic experiences with caregivers later on in childhood interact with — and have the potential to gradually update — these early schemas. Higher caregiving quality at ages 8 and 12 is therefore likely to be associated with more episodic experiences of support in the contexts of school-related and other child activity challenges, and updated scripts thereby, eventually modifying also one’s expectations of sensitive support. Thus, it is perhaps not surprising that the strongest links are between caregiving behavior in late childhood and adolescence and the adolescents’ secure base scripts, especially given the frequent changes in caregiving environments that many of these children experienced. These findings suggest that interventions aimed at enhancing caregiving quality during middle childhood may be particularly fruitful in promoting secure attachment in adolescence.

### Limitations

One notable limitation of the current study is the relatively small sample size, which could limit the power to detect more subtle group differences. In addition, given that the study participants experienced early deprivation, the pattern may be different when considering other forms of maltreatment (e.g., abuse). Future research should aim to replicate these analyses with larger samples to strengthen the validity and generalizability of the observed associations. Second, the reliance on the secure base scripts as the primary measure of attachment, while valuable, may have inherent limitations. Assessing attachment beyond early childhood is challenging because it becomes more abstract and most commonly relies on indirect measures such as the prompted structured narratives used in the present study. Secure base scripts, typically assessed through prompt-word story tasks, are thought to reflect mental representations of attachment, though the extent to which they reliably capture real-world relational dynamics is debated. Some researchers note that these tasks may conflate storytelling skill with attachment knowledge or overlook contextual influences (Kerns et al., 2011; McLean et al., 2014). Nevertheless, research shows secure base script scores predict social competence, stress regulation, and memories of caregiver support, even after controlling for verbal ability (Dykas et al., 2006; Psouni & Apetroaia, 2014; Psouni et al., 2015; Steele et al., 2014). However, because the Secure Base Script Test reflects a generalization of attachment experiences over time, it cannot separate early attachment representations from later developments (or changes) as a result of new experiences with (other different) caregivers. Thus, including other measures, like reflective functioning or adolescents’ ability to provide coherent narratives about attachment, might have enhanced our ability to capture differences between groups such as the FCG and CAUG.

Third, retention challenges, particularly in the NIG, pose a limitation. Higher attrition in this group likely reflects the study’s long duration and family mobility. Many NIG participants entered at age 8, and a substantial portion discontinued by adolescence. To minimize group contamination, we excluded NIG participants added at age 8 from analyses. Finally, although our methods for parental quality assessment demonstrated high reliability, it is possible that the different assessments of caregiving in early and later childhood may have affected results.

## Conclusion

This is the first study to examine the impact of the deprivation inherent in institutional rearing on secure base scripts knowledge among adolescents. Our findings attest to that early institutional care interferes with the development of secure base scripts knowledge that can be captured even later in adolescence, as also evidenced by lower secure base scripts scores in previously institutionalized groups compared to their non-institutionalized peers. Despite predictions that the foster care intervention to mitigate these effects, our findings suggest that the complexities of attachment dynamics may not be easily altered by such interventions alone. Importantly, however, our study sheds light on the influence of caregiving quality over time interacting with scripted knowledge, underscoring the importance of continued support and interventions in improving caregiving quality well into the middle childhood and pre-adolescent years, to foster better developmental outcomes in terms of attachment into adolescence.

## Supplementary Information

Below is the link to the electronic supplementary material.

ESM 1 (DOCX 17.6 KB)

## Data Availability

Data available upon request.
