# Supplementary material for: Caregiving Quality Across Development and Secure Base Knowledge among Adolescents with a History of Institutional Care
Source: Advers Resil Sci. 2025 Oct 26;6(4):603–12. doi: 10.1007/s42844-025-00183-2 (PMC12660420; doi:10.1007/s42844-025-00183-2)
Supplement: Supplementary file 1 — (DOCX 17.6 KB) [file 42844_2025_183_MOESM1_ESM.docx]

Supplemental Table S1. High and low score narratives from the “Accident” and “Troubles at school” storylines

| ***Accident*, higher range score** |
| --- |
| On her birthday, Ioana received a very beautiful new bike as a gift from her parents. It was as she wanted it, blue, full of very beautiful artistic details. But one day, not being prepared for her new bike’s speed, Ioana had an accident, she got hit - not very seriously, but still - pretty bad. She was crying. Her mother and father went to her; they took her to the doctor. The doctor put a bandage on her while her mother and father tried to make her feel better. Her mother took her in her arms and told her that everything was going to be fine, and she felt better. She was not hurting so much after a while, so her parents took her home, and her father fixed the bike. Then her cousins came to the house and gave her support – they told her to move on, that it was an accident and it will not happen again if she is careful, and they also brought her gifts. They all had dinner together. |
| ***Accident*, middle range score** |
| Ionut’s mother and father bought him a new bike. He is very excited about this bike, he speed-cycles up the hill and falls off the bike and gets hurt. Ionut starts crying, he goes to his mother and father, they see that he’s wounded, they take him to the doctor’s, the doctor bandages Ionut’s hand. His father takes him in his arms and tells him not to worry, everything will be fine, meanwhile his grandfather is at home fixing the broken bike, then they all go home and have dinner. |
| ***Accident*, lower range score** |
| It happened during a rainy day. Ionut went for a ride in town with his bicycle. He stumbled on a stone and fell. Upon hearing this, his mother and father started crying. “Ionut, come home …” And he came home with a broken arm. They called the doctor. The doctor noticed that he didn’t have one broken arm but both arms were broken, so the doctor took him to the hospital. At the hospital he was told: “Your left arm is broken, and your right arm is broken too.” The doctor asked: “How could the accident happen? Next time don’t cycle with such speed when it’s raining and especially when there are stones on the ground, Ionut.” He had cast applied on both arms. |
| **Troubles at School, higher range score** |
| Ioana was in the 6^th^ grade, and Alexandra was her best friend, and she was also her desk mate. They got along very well, they had many things in common, they visited one another, they went out and played and one day, during a break, they went on the hallway because someone called them. And next to them there were a few older children, 7th or 8th graders who pushed Ioana. They said they had done it as a joke, but actually it had been on purpose, Alexandra realized, and she was furious because they had hit her best friend. She defended Ioana and told the older ones to pick on someone their own age who can defend themselves and not a girl who never did anything to them. The older children teased and made fun of Alexandra, but then the bell rang. Ioana was very grateful to Alexandra because she was such a good friend, and she realized she was going to be there to help and support her in the more difficult moments such as this one. They continued playing and they felt proud of having been able to defend themselves. |
| **Troubles at School, middle range score** |
| Ioana and Alexandra became good friends in the second day of school. During a break, some older kids, 8^th^ graders, pushed Alexandra and Ioana angrily defended her. Ioana made the 8^th^ graders feel embarrassed and leave and let Alexandra be. The older children left and Alexandra was very grateful to Ioana, then they started playing, until the break was over. |
| **Troubles at School, lower range score** |
| So the older children didn’t like Ionut and the others… they ran because they were afraid, the gang were bigger, and when the gang gathered, they started running scared… there was a fight. When the fight began, they called that guy and said: “Man, come here because some guys picked on me and they want to hit my son at his school”. And when they came to school the following day, they made a scene with their other mates. After they talked about all this, they formed a friendship and each went on his way. |

| Supplemental Table S2. Spearman Correlations Between Caregiving Quality and Secure Base Script Test at age 16 | | | | | | |
| --- | --- | --- | --- | --- | --- | --- |
|  | 1 | 2 | 3 | 4 | 5 | 6 |
| 1. Caregiving Quality 30 months | 1 |  |  |  |  |  |
| 2. Caregiving Quality 42 months | .34*** | 1 |  |  |  |  |
| 3. Caregiving Quality 54 months | .33*** | .29** | 1 |  |  |  |
| 4. Caregiving Quality 8 years | .39*** | .28** | .83*** | 1 |  |  |
| 5. Caregiving Quality 12 years | .39*** | .26** | .74*** | .84*** | 1 |  |
| 6. SBST age 16 years | -.03 | -.06 | .20* | .22* | .23* | 1 |
| *Note*. SBST = secure base script test. * *p*<.05. ***p*<.01. ****p* <.001. | | | | | | |
